# The effect of different carbon reductants on the production of ferrosilicon 75% on an industrial scale in an electric arc furnace

**DOI:** 10.1016/j.heliyon.2023.e13956

**Published:** 2023-02-24

**Authors:** Alireza Etemadi, Hassan Koohestani, Mohammad Tajally

**Affiliations:** Faculty of Materials and Metallurgical Engineering, Semnan University, Semnan, Iran

**Keywords:** Ferrosilicon, Carbothermic reduction, Reducing agents, Semi-coke, Fixed carbon

## Abstract

Ferrosilicon production is based on the carbothermal reduction of silica and iron oxide in submerged electric arc furnaces. The reducing process of iron oxide and silicon oxide is carried out by the carbon contained in carbon materials such as coal, charcoal, semi-coke, and all types of coke. Based on its inherent and functional characteristics, the kind of carbon material can be effective in the ferrosilicon production process and furnace energy consumption. Therefore, in this work, which was performed by Iran Ferrosilice company, in a long period of 5 years, the effects of seven combinations of different carbon materials on the electrical and metallurgical performance of the process were investigated. The results showed that the minimum value of energy coefficient per ton (8.46 MWh/ton) was obtained using combination 5 (consisting of 55% coal, 30% semi-coke, 5% charcoal, and wood chips). The use of wood chips reduced the energy consumption by 3.03 MWh/ton. The composition consisting of 50% coal, 35% semi-coke, 15% charcoal, and wood chips had the highest Si% of 73.64% and the lowest Al% of 1.54%. Finally, by evaluating all the results, especially the reduction of energy consumption and recovery of Si, compound 5 was introduced as the optimal compound in the ferrosilicon production process.

## 1 Introduction

Ferroalloys are compounds containing iron and other elements, mainly used in steelmaking. Ferrosilicon, the most consumed ferroalloy, is used for alloying, nucleating, and deoxidizing. It is produced in electric arc furnaces based on the carbothermic reduction of silica (quartz) and iron oxide. Carbon materials play a very significant role as a reductant [1,2].

The electricity consumption of electric arc furnaces is in the range of 7000–8000 kWh per metric ton of ferroalloy. In this case, the higher electrical resistance of the charge material is necessary for heating the hearth of the electric furnace. This way, the electrode tip positions to become deepen in the furnace, which can reduce the current passing through the charge by about one-third [3,4]. Therefore, in the ferrosilicon production process, attention to two factors is of particular importance: first, the presence of carbon materials as reducing agents, and second, the very high electric energy consumption by the arc furnace to break the bonds of elements in oxides. Various carbon materials are used, including coal, char, metallurgical coke, petroleum coke, graphite, charcoal, wood, and semi-coke [1,5,6].

The selection of reducing agents for the ferrosilicon production process requires consideration of several criteria, such as economic considerations, appropriate chemical composition (low ash, high reactivity, low volatile substances, high fixed carbon, etc.), high electrical resistance, appropriate granulation, and mechanical strength is high. Due to the importance of carbon materials in the production of ferroalloys, various studies have been conducted on these materials as reducing agents in the process [1,3,7].

The reactivity against oxidation and the specific electrical resistance of carbon materials are the most significant reducing parameters used in the sub-charge arc furnace. In general, the specific electrical resistance decreases with the increase of graphitization level. Also, the presence of carbon atoms in the amorphous phase or fatty side chains increases reactivity [[8], [9], [10]]. The reactivity of carbon materials affects the consumption of electrodes and the life of refractories by affecting the atmosphere of the furnace [11]. As a result, the rate of combustion or conversion to gas is very different for various reducing agents. With the progress of combustion, due to the reduction of volatile substances, the improvement of the thermal annealing process, and the decline of the concentration of active sites, the reactivity decreases. In addition to structural order and physical properties (such as pore size distribution and surface areas), the composition and concentration of ash impurities in the carbon material also play an essential role [1,3,6].

The most significant role of carbon materials is to react with SiO gas to convert it into SiC. The ability of reductant agents to react with SiO gas is recognized as its SiO-reactivity. This factor is one of the main parameters in the melting process to achieve a high yield of metal [3,12]:

(1) $$SiO_{\left(g\right)}+2C_{\left(s\right)}=SiC_{\left(s\right)}+CO_{\left(g\right)}$$

Therefore, a solid silicon carbide layer is formed on the surface, which can block the reaction surface and reduce the reactivity of SiO over time [5]. About 80% of the silicon in the gas phase is recovered to produce ferrosilicon, and 20% of the SiO gas is discharged by the off-gases [7].

Compared to coke, semi-coke, and industrial coal, charcoal has lower thermal abrasion strength, low sulfur, and phosphorus content, high humidity, low mechanical strength, higher CO_2_-reactivity and higher electrical resistivity. Of course, charcoal has a lower fixed carbon content and much more volatile substances than them [1,3,13,14]. Charcoal particles are completely isotropic and have many active sites to react with CO_2_. Therefore, the addition of charcoal increases its CO_2_-reactivity due to the reduction of the ratio of anisotropic texture in coke [15].

The mechanical properties of carbon materials are very significant for use in ferroalloy industries. Because high mechanical strength is needed to prevent the collapse of the carbon bed by load, and to minimize the production of fine particles during the handling and feeding of charcoal particles to the reactor, it is necessary to have a suitable abrasive strength [7].

Pavlov et al. [12], with direct dilatometric measurements in simulated reducing processes in ferroalloy furnaces, showed that based on the progress of the process, coal has more reaction power than semi-coke and coke. But coal is mechanically destroyed under a load of 2 kg/briquette, at 500–700 °C [16,17].

Suharno et al. investigated the effect of coke and coal as reducing agents on the quality of ferromanganese, especially considering the content of manganese, sulfur and phosphorus, and other parameters. Compared to coal, the application of coke as a single reductant agent, led to a higher percentage of manganese content and a higher amount of ferromanganese product. By using coke, higher efficiency and lower specific energy of the ferromanganese production process were obtained. Using coal as a reducing agent compared to coke leads to ferromanganese containing more phosphorus and sulfur [18].

Due to the high dissolution rate of coke in liquid metal, its slag reaction (slag-carbon reaction) is faster than charcoal. However, the highest charcoal reaction rate was reported in the charcoal samples without alkaline lean [14,19]. Among various cokes, coke with lower reactivity may create weak reducing conditions and large coke beds in the furnace, as a result, poor furnace control, unstable slag, and more energy consumption will follow [20]. Recently, it has been shown that the use of biomass in the coal mixture for coke production reduces the cost and CO_2_ emissions [15].

A mixture of different carbon materials plays a very significant role in the energy consumption and efficiency of the exergy process. Among these materials, the preparation of charcoal requires large amounts of wood, and continuous and widespread deforestation leads to ecological damage. Therefore, the study of alternative carbon reductants of charcoal, by finding strategies that do not require the usage of charcoal in silicon production processes, is an urgent demand [5,7,17].

In ferroalloys production, the carbon material used has a great impact on the overall process cost, energy consumption, operation stability and final product quality [21]. So far, a comprehensive study on the effect of different combinations of carbon materials for the actual process of ferrosilicon production on an industrial scale has not been done. Therefore, in this work, the production process of ferrosilicon has been evaluated for a long period of time in Iran Ferrosilice Co. For this aim, according to the different properties of carbon materials (including ash, fixed carbon, volatile materials, and moisture), the effect of carbon materials combinations (containing different amounts of wood chips, charcoal, coal, coke, semi-coke) on the important metallurgical and electrical parameters of the electrical arc furnace and production process has been studied. Comparing the results, the best combination of carbon materials that have had optimal results on an industrial scale is introduced.

## 2 Materials and methods

This work was done in the Iran Ferrosilice Co. (Semnan privacy), which has a sub-charged electric arc furnace with a production capacity of 25,000 tons per year and an apparent power of 55.2 MVA. The furnace control is done by the Minstral control system, which is based on the resistance of the electrodes and is done automatically. This furnace is semi-open and includes three Soderberg-type electrodes with a diameter of 145 cm. An electric arc furnace is a rotary furnace that performs continuous melting.

In this research, seven different carbon compounds containing metallurgical coke, coal, charcoal, semi-coke, and wood chips were prepared according to Table 1. Carbon materials and quartz were first crushed in a cone crusher and a ball mill until their particle sizes were 5–25 mm and 25–80 mm, respectively. Carbon materials with constant weight amounts of quartz (750 kg) and rolling rustling (120 kg) were entered into the furnace, maintaining the following ratio:

(2) $$\frac{\begin{array}{cc} fixed & carbon \end{array}}{SiO_{2}}=0.4$$

**Table 1:** components of various compounds used.

Kg	Wood chips	Charcoal	Coal	Semi-coke	Coke
COM1	180	0	315	130	50
COM2	0	0	280	115	50
COM3	180	0	230	180	50
COM4	0	100	270	185	0
COM5	180	40	340	130	0
COM6	180	100	270	150	0
COM7	180	100	240	100	50

Granulated raw materials enter the furnace from the upper part of the furnace through multiple pipes. The necessary heat for the endothermic reaction of silica reduction is provided by creating an electric arc at the tips of the electrodes. About 75 tons of ferrosilicon melt is continuously produced daily.

**Determination of moisture content:** 10 g of coal and charcoal for 60 min at 105 °C, and coke and coke for 240 min at 200 °C were heated. Then the percentage of moisture (M) was obtained according to the difference in weight:

(3) $$\%M=\frac{\text{amount}\;\text{of}\;\text{weight}\;\text{loss}\;\left(g\right)}{\text{initial}\;\text{weight}\;\text{of}\;\text{sample}\;\left(g\right)}\times 100$$

**Determining the amount of ash:** 1 g of dried sample was heated at 815 °C for 60 min to burn its carbon content. Then the percentage of ash (A) was calculated:

(4) $$\%A=\frac{\text{weight}\;\text{of}\;\text{ash}\;\left(g\right)}{\text{initial}\;\text{weight}\;\text{of}\;\text{sample}\;\left(g\right)}\times 100$$

**Determining the amount of volatile materials:** 1 g of sample was heated at 90 °C for 7 min, and the percentage of volatile materials (VM) was calculated based on the difference in weight:

(5) $$\%\text{VM}=\frac{\text{amount}\;\text{of}\;\text{weight}\;\text{loss}\;\left(g\right)}{\text{initial}\;\text{weight}\;\text{of}\;\text{sample}\;\left(g\right)}\times 100-M$$

**Determining the amount of fixed carbon:** The fixed carbon (FC) of dry samples was calculated with the following equation:

(6) %FC=100−(%A−%VM)

### 2.1 Chemical analysis of quartz

The chemical analysis of quartz used for different compositions of carbonaceous materials is presented in Table 2. The details of carbon material analysis (%fixed carbon, % moisture, %ash, and %volatile materials) are also shown in Table 3.

**Table 2:** Average chemical composition of quartz used in different combinations of carbon materials.

%wt	SiO2	Al2O3	Fe2O3	CaO	MgO
COM1	96.97	0.92	0.77	0.49	0.14
COM2	96.48	0.87	1.34	0.31	0.11
COM3	97.90	0.70	0.49	0.36	0.06
COM4	97.74	0.57	0.46	0.56	0.20
COM5	97.84	0.62	0.64	0.34	0.10
COM6	98.05	0.57	0.44	0.20	0.06
COM7	98.29	0.35	0.50	0.22	0.04

**Table 3:** Details of the analysis of the carbon materials used.

%	Fixed carbon	moisture	ash	volatile materials
Coal	53–60	3–10	8–12	30–33
Charcoal	75–78	5–13	8–10	13–16
Coke	81–87	1–3	11–16	2–5
Semi-coke	85–87	7–14	5–9	7–8

Recording of quality conditions and analysis of the product every day has been done with three sampling times, each of which was the average of three analyses. All raw materials were analyzed repeatedly when used.

## 3 Results and discussion

The percentage of reducing materials based on the amount of fixed carbon is presented in Table 4.

**Table 4:** The percentage of carbon materials based on the amount of fixed carbon in the compounds.

	%fixed carbon				kg
	Charcoal	Coal	Semi-coke	Coke	Wood chips
COM1	0	50	35	15	180
COM2	0	55	30	15	0
COM3	0	40	45	15	180
COM4	15	40	45	0	0
COM5	5	65	30	0	180
COM6	15	50	35	0	180
COM7	15	45	25	15	180

Production and electrical parameters of ferrosilicon production by different combinations of carbon materials were evaluated in 30-day periods, and their average is presented in Table 5.

**Table 5:** the average value of the important parameters in the ferrosilicon production process.

	production tonnage (ton)	energy consumption (MWh)	energy per ton (MWh/ton)	%Si in the product	%Al in the product	Voltage (v)
COM1	2339	20497	8.76	72.71	1.91	437.5
COM2	2358	20491	8.69	73.25	1.77	423.6
COM3	2315	20353	8.79	73.11	1.80	413.5
COM4	2146	19696	9.18	73.08	1.68	411.7
COM5	2160	18175	8.46	73.62	1.55	423.2
COM6	2336	20385	8.73	73.64	1.54	436.8
COM7	2274	19589	8.61	72.99	1.73	412.2

**Fig. 1:**
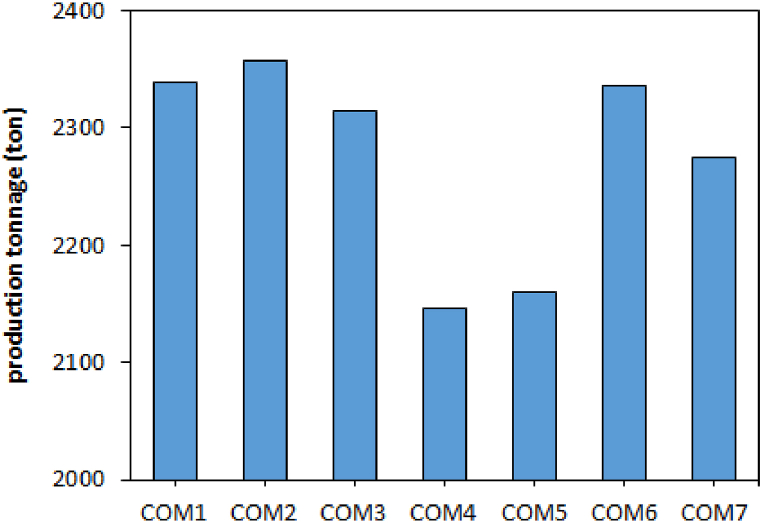
The amount of production tonnage using different combinations of carbon materials.

Fig. 1 shows the product yield when using different combinations of carbon materials. The highest and lowest production rates were achieved using COM2 (2358 tons) and COM4 (2145 tons), respectively. The reaction between SiO2 and 3C consists of two reactions, the reaction between SiO2 and C:

(7) $$SiO_{2\left(s\right)}+C_{\left(s\right)}=SiO_{\left(g\right)}+CO_{\left(g\right)}$$

And the reaction between SiO and 2C [22]:

(8) $$SiO_{\left(g\right)}+2C_{\left(s\right)}=SiC_{\left(s\right)}+CO_{\left(g\right)}$$

The weight reduction of charge particles is affected by 1) the rate of SiO gas production by the reaction (7), and 2) the rate of SiO gas absorption by the reaction (8). The reaction rate of these two reactions is affected by the quartz/carbon reaction and the reactivity of SiO, respectively. The higher the reactivity, the faster the reaction [23]. The general rate of reactivity of carbonaceous materials with SiO2 is charcoal > coal > metallurgical coke > pet coke [3]. Coke with higher mechanical strength is more reactive than other carbon materials [15].

**Fig. 2:**
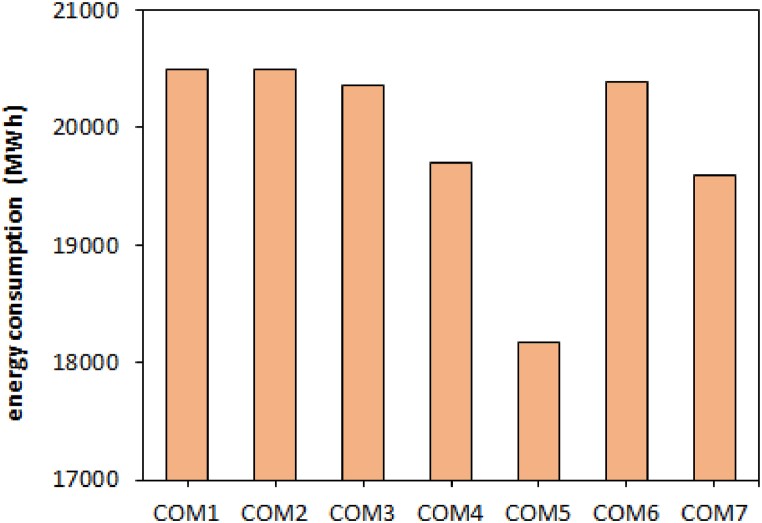
Energy consumption using different combinations of carbon materials.

Since the amount of energy consumption directly affects the production value, both parameters were simultaneously considered to evaluate the effect of carbon materials. Fig. 2 shows the amount of energy consumption when using different carbon compounds. The lowest and highest energy consumption was obtained using COM5 (18175 MWh) and COM1 (20497 MWh), respectively.

**Fig. 3:**
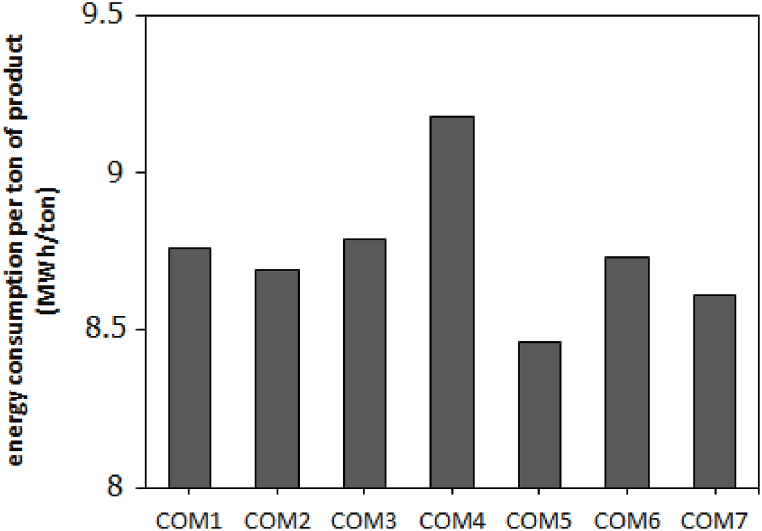
Energy consumption per ton of produced ferrosilicon for various carbon compounds.

The energy factor per ton shows the energy consumption per ton of product, which is obtained by dividing the consumed energy by the production tonnage (Table 4 and Fig. 3). By comparing this factor, the effect of compounds can be investigated more precisely. The results show that the lowest value of this factor was obtained using COM5 (8.46 MW/ton).

Comparing COM1 and COM2, the addition of wood chips changed the energy factor from 8.76 to 8.69, which is not significant. In COM4 and COM3, where charcoal was used instead of coke, the energy factor increased from 8.79 to 9.18, and charcoal was not a good substitute for coke. Of course, the presence of wood chips in COM3 has had a positive effect on reducing the energy factor.

COM3 and COM4 have more half-coke content than COM2. Semi-coke compared to coke, has higher energy consumption due to volatile substances and high humidity. To understand this issue, COM1 can be compared with COM5. In these two samples, the amount of semi-coke and wood chips is the same. In COM5, coal was used instead of coke, which reduced energy consumption. On the other hand, the use of carbon material with high CO_2_-reactivity, such as charcoal, causes the expansion of the combustion reaction and thus increases the consumption of carbon and energy. Therefore, the ideal carbon material should have low CO_2_-reactivity [21,21].

Meanwhile, charcoal has a higher electrical resistance that decreases with increasing temperature, and retention time [15,19]. If the reduction of iron oxides with CO occurs at a temperature where the Boudouard reaction is active, the produced CO_2_ reacts with the carbon materials. Since this is an endothermic reaction, it increases energy consumption and carbon consumption [21].

In COM5, reducing the amount of semi-coke and increasing the amount of coal has caused a significant decrease in the energy coefficient. In addition, the presence of wood chips has a positive effect on improving the energy factor. Therefore, the high reactivity of semi-coke is explained by the greater porosity and internal specific surface area of the material compared to coke and especially graphite (Apparent porosity of semi-coke 12.58% and coke 3.37%) [24]. Semi-coke has a highly branched internal structure and is even more than the original coal. Due to the eruption of excessive volatiles, coal breaks easily. Therefore, it provides greater reactivity than semi-coke. However, the production of a large number of fine particles in the inlet package of the electric furnace reduces the gas permeability and disrupts the natural flow of the reductant process [17].

The results showed that to improve Si recovery, reduce energy consumption and improve production, the amount of coal should be increased, and the amount of semi-coke should be decreased. If in COM6, the amount of coal and semi-coke has increased.

Due to the higher porosity of the semi-coke, the transfer of SiO gas from the particles is easy and increases its reactivity and furnace efficiency. In addition, at all temperatures, the specific electrical resistance of semi-coke is more than metallurgical coke [24]. Therefore, the presence of semi-coke increases Si recovery, improves furnace efficiency, and reduces electrical energy consumption [24]. Comparing COM6 with COM1, where charcoal is used instead of coke, the energy factor is slightly reduced. Although the change was insignificant, the results obtained are entirely reasonable due to the superior properties of charcoal over metallurgical coke as a reductant.

Comparing the reactivity of carbon materials in quartz reduction, Li et al. [18] showed that charcoal has a good performance in both SiO–C reactivity and quartz/carbon reaction, and it should be used as the principal carbon material in the Si production process. Coke is effective for absorbing SiO gas. In general, coal is not an ideal carbon material for quartz reduction in the Si production process [23]. The results show that by using 50–55% coal and 30–35% coke, the amount of energy consumption is minimized.

In COM7, all carbon materials are used along with wood chips. In this way, the effect of the simultaneous use of all available carbon materials can be determined on quantitative factors (energy consumption and production tonnage) according to the different characteristics of each. According to the energy factor value of 8.61 MWh/ton for COM7, it can be concluded that due to the diverse physical, chemical, and technological properties of different carbon materials, each of these materials is considered a reductant in various parts of the furnace and diverse thermal and electrical conditions. The simultaneous use of all of them will play a significant role in the optimization of quantitative parameters in the ferrosilicon production process.

### 3.1 silicon recovery

The amount of silicon recovery is a function of the position of the electrode tip (electrode position), the amount of fixed carbon available, the reactivity of reducing agents, the electrical strategy, the quality of quartz, etc. But the main factor in determining the amount of Si recovery is the behavior of the reducing agents (including reactivity, granulation, thermal shock resistance, etc.) in the furnace. Therefore, its evaluation in the application of each of the carbon compounds determines their reduction performance. Silicon recovery is calculated from the following equation [ [17,25]]:

(9) $$\text{Si}\;\text{recovery}=\frac{\text{Si}\;\text{in}\;\text{the}\;\text{product}}{\text{Si}\;\text{in}\;\text{silica}\;\text{mineral}}\times 100$$

**Fig. 4:**
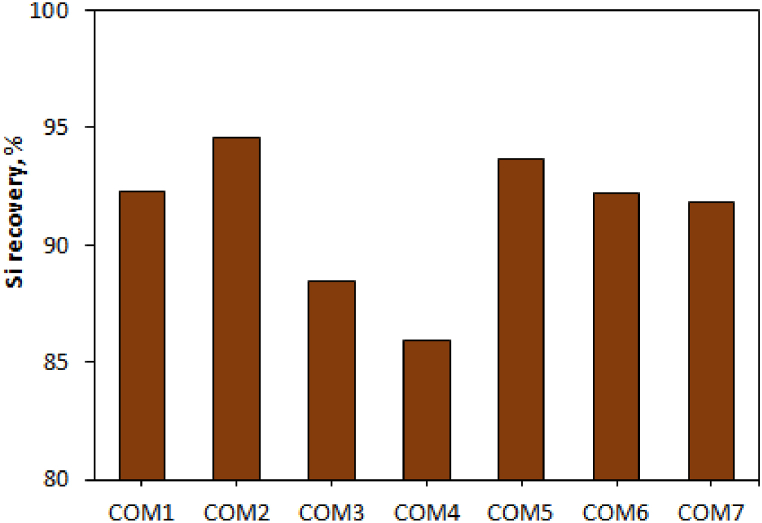
Silicon recovery when using various carbon compounds. (For interpretation of the references to colour in this figure legend, the reader is referred to the Web version of this article.)

The average %Si of the quartz mineral was obtained from its monthly analysis. While the average Si of the product was determined from the daily analysis (during three days and every day in 3 working shifts) (Table 6 and Fig. 4).

**Table 6:** Silicon recovery percentage and silica mineral consumption when using different carbon compounds.

	COM1	COM2	COM3	COM4	COM5	COM6	COM7
% Si recovery	92.25	94.55	88.48	85.90	93.68	92.21	91.85
silica mineral consumption (ton)	4072.2	4057.5	4187.6	4002.2	3716.7	4119.3	3940.3

In the ferrosilicon process, SiO gas produced at high temperature is captured by carbon materials at low temperature and is converted into β-SiC according to the reaction (3). If the carbon material cannot capture SiO, it will oxidize to SiO2 and escape from the furnace. In this case, because the production of SiO is highly endothermic, in addition to reducing of ferrosilicon production (recovery of Si), it increases energy consumption. The most important factor affecting energy consumption is the reactivity of SiO with carbon material [ [21,26]].

The kinetics of the reaction depends on the particle conditions containing size, amount of porosity, type of porosity and morphology. This reaction is also very dependent on the carbon material used. Unlike coal, metallurgical coke and petroleum coke have low SiO reactivity. Of course, the production method of carbon material (feedstock and production conditions) must be considered. For example, Charcoals produced at lower heating rates have higher SiO reactivity [ [27,28]].

The best and worst performance was related to COM2 (94.55%) and COM4 (85.9%), respectively. The compounds that use less energy have better Si recovery. In other words, the best recovery and the best results of quantitative parameters were related to the use of COM5 and COM2, and the weakest results were in the use of COM3 and COM4.

The most important influencing parameters of carbon materials on Si recovery are porosity, electrical resistance, and percentage of aluminum oxide in ash. These parameters are affected by other factors [24]. For example, the amount of porosity and temperature affect the electrical resistance. According to Tian-ming et al.'s model, with increasing porosity and increasing temperature, the electrical resistance of carbon materials increases [29]. Electrical resistance decreases with increasing temperature due to thermal expansion in carbon materials. The higher the porosity, the more thermal expansion occurs [ [30,31]].

In COM6 compared to COM4, in which wood chips are added, lower energy consumption, higher recovery, and higher production rate have been obtained, which shows the positive effect of wood chips on process performance.

### 3.2 Consumable voltage and volatile materials

To provide an efficient reduction environment, thermal energy must be produced at the tips of the electrodes. The proper practical method to create these conditions is to supply a voltage reductant or to use materials with high electrical resistance. Because low voltages lead to a decrease in furnace power and efficiency, the use of charging materials with higher electrical resistance can be done without disturbing the process. On the other hand, the specific electrical resistance of carbon materials decreases with the increase of carbon content and increases with the excess of volatile materials. The electrical resistance of metallurgical coke decreases from 13 mΩ m at 1000 °C to about 6.5 mΩ m at 1600 °C for the particle size of 4.75–9.5 mm [25]. While for charcoal particles larger than 2 mm up to about 50 mΩ m at 1100 °C and 17 mΩ m at 1600 °C have been measured [14].

**Fig. 5:**
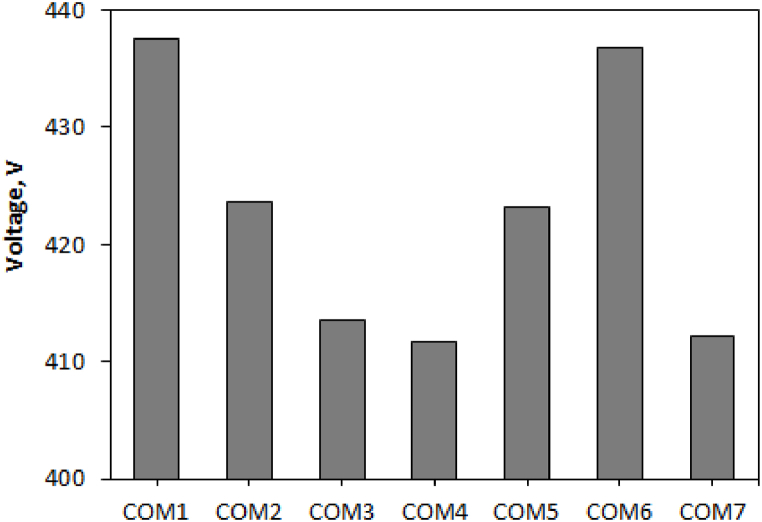
The amount of volatile substances in various carbon compounds.

The furnace voltage changes for different combinations of carbon materials are shown in Fig. 5. Increasing the electrical resistance of the charge makes it possible to use of higher voltages, which can improve the power and efficiency of the furnace. Based on the penetration depth of the electrodes and the generation of thermal energy at the tip of the electrodes, COM1, 5, and 6 create more successful reduction conditions.

**Fig. 6:**
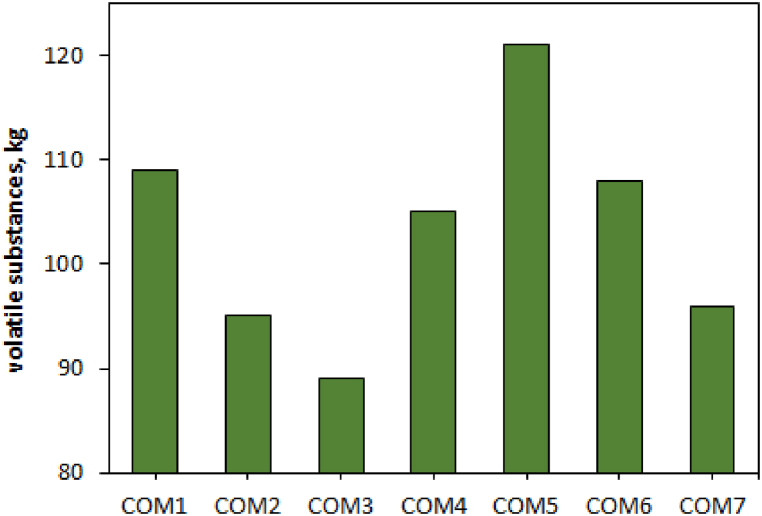
The amount of volatile substances in various carbon compounds.

All compounds had the same amount of fixed carbon (due to the same ratio of $\frac{C_{fix}}{SiO_{2}}$). However, COM5 and COM3 have the highest and lowest amount of volatiles, respectively (Fig. 6). The comparison of furnace voltage using different combinations shows that the highest and lowest voltage values correspond to COM1 (highest charging resistance) and COM4 (lowest charging resistance), respectively.

### 3.3 %Si of the product

**Fig. 7:**
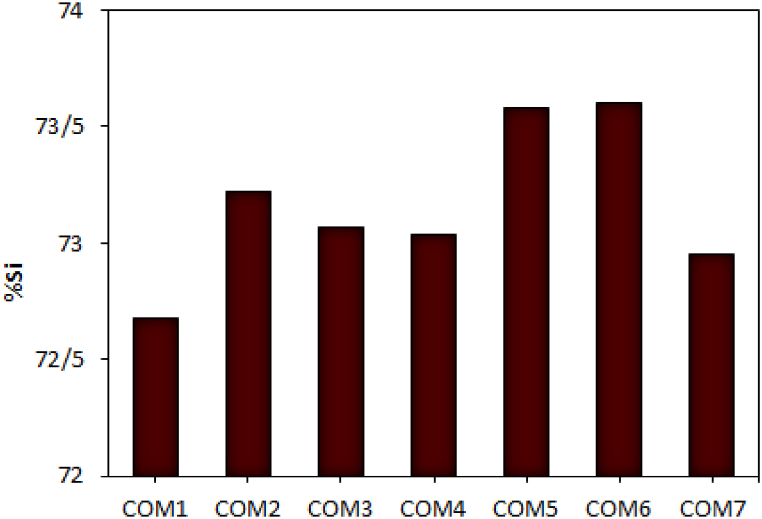
The average percentage of silicon in the product using various carbon compounds. (For interpretation of the references to colour in this figure legend, the reader is referred to the Web version of this article.)

The ferrosilicon produced by Iran Ferrosilice Co. is 75% ferrosilicon (containing 75-72% Si). The amount of silicon in different samples in Fig. 7 shows the appropriate result for COM5 and COM6. The lowest amount of silicon was obtained in COM1.

## 4 Conclusion

Optimum energy consumption in the ferrosilicon production industry has caused special attention to the raw materials used, especially carbon materials. Usually, the combination of carbon materials is used as a reductant. Therefore, in this study, the effect of different combinations of carbon materials on the ferrosilicon production process in Iran Ferrosilice Company was investigated in a 5-year period. Combinations of various carbon materials containing charcoal, coal, wood chips, coke, and semi-coke were used maintenance the ratio $\frac{\begin{array}{cc} fixed & carbon \end{array}}{SiO_{2}}=0.4$. Fixed carbon content, porosity, SiO-reactivity, and CO_2_-reactivity are among the characteristics of carbon materials that can affect the electrical and metallurgical performance of the furnace. The results showed that the use of a combination of 65% coal, 30% semi-coke, 5% charcoal, and wood chips has a good performance in the production process of ferrosilicon and can be recommended as the optimal carbon combination. With this combination, the lowest amount of energy consumption per ton of ferrosilicon production (8.46 MWh/ton), the appropriate Si recovery rate (93.68%), and the high %Si in the product (73.58%) were obtained. It is possible to introduce an optimal combination of carbon materials by studying the thermodynamics and kinetics of process reactions with the characterization of raw materials.

## Author contribution statement

Alireza Etemadi: Conceived and designed the experiments; Performed the experiments; Contributed reagents, materials, analysis tools, or data; Wrote the paper.

Hassan Koohestani: Conceived and designed the experiments; Analyzed and interpreted the data; Contributed reagents, materials, analysis tools, or data; Wrote the paper.

Mohammad Tajally: Conceived and designed the experiments; Analyzed and interpreted the data; Contributed reagents, materials, analysis tools, or data.

## Funding statement

This research did not receive any specific grant from funding agencies in the public, commercial, or not-for-profit sectors.

## Data availability statement

No data was used for the research described in the article.

## Declaration of interest’s statement

The authors declare no conflict of interest.
